# Fundamental and Applied Advances in Stem Cell Therapeutic Research

**DOI:** 10.3390/cells11121976

**Published:** 2022-06-20

**Authors:** Makram Merimi, Saida Rahmani, Ahmed Afailal Tribak, Fatima Bouhtit, Hassan Fahmi, Mehdi Najar

**Affiliations:** 1 Experimental Hematology, Jules Bordet Institute, Unive and nd nd ité Libre de Bruxelles, 1070 Bruxelles, Belgium; makram.merimi.cri@gmail.com (M.M.); saida.ramani@gmail.com (S.R.); tribakafailal.ahmed@gmail.com (A.A.T.); bouhtitfatima@gmail.com (F.B.); 2 Genetics and Immune Cell Therapy Unit, Faculty of Sciences, University Mohammed Premier, Oujda 60000, Morocco; 3 Osteoarthritis Research Unit, University of Montreal Hospital Research Center (CRCHUM), Department of Medicine, University of Montreal, Montreal, QC H2X 0A9, Canada; h.fahmi@umontreal.ca; 4 Laboratory of Clinical Cell Therapy, Jules Bordet Institute, Université Libre de Bruxelles, 1070 Brussels, Belgium

We are pleased to present this Special Issue of *Cells*, entitled ‘Feature Papers in Stem Cells’. We hope that this collection of papers may contribute greatly to this field by discussing and presenting new outcomes of basic and translational stem cell-based regenerative medicine research. The rapid progress in the field of stem cell research has laid strong foundations for their use in regenerative medicine applications involving injured or diseased tissues. Cellular therapy aims to replace damaged resident cells by restoring cellular and molecular environments suitable for tissue repair and regeneration. Growing evidence indicates that some of the observed therapeutic outcomes of stem cell-based therapy are due to paracrine effects (including extracellular vesicles), rather than long-term engraftment or the survival of transplanted cells [1]. Embryonic and induced pluripotent stem cells (ESCs and iPSCs), as well as adult stem cells, hold great promise for future cell replacement therapies. Among other candidates, mesenchymal stem/stromal cells (MSCs) represent a critical component of stromal niches known to be involved in tissue homeostasis [2]. Additional evidence suggests that MSCs originate from perivascular cells—principally pericytes that are vascular mural cells—within multiple human organs including lung, adipose tissue and placenta [3]. Accordingly, MSCs play a crucial role during lung development by interacting with the airway epithelium, and also during lung regeneration and remodeling after injury, particularly in chronic obstructive pulmonary disease [4]. During tissue healing, MSCs may exhibit several therapeutic functions to support the repair and regeneration of injured tissue. The process underlying these effects likely involves the migration and homing of MSCs, as well as their immune-tropic functions [5]. Interestingly, tissue-nonspecific alkaline phosphatase (ALP) (TNSALP), a ubiquitous membrane-bound glycoprotein capable of providing inorganic phosphate by catalyzing the hydrolysis of organic phosphate esters, or removing inorganic pyrophosphate that inhibits calcification, is highly expressed in juvenile cells, such as pluripotent stem cells (i.e., ESCs (iPSCs) and somatic stem cells (i.e., MSCs), and is involved in their maintenance and differentiation [6]. Understanding and controlling these cellular products requires in-depth knowledge of their maintenance mechanisms and their exit from undifferentiated states in specific biomaterials mimicking native niches. An interesting approach has been established for differentiating porcine epiblast stem cells (pEpiSCs) into proliferating and functional endothelial cells (ECs). Functional tests revealed that the generated ECs could be used in in vitro assays to examine angiogenesis or cellular responses to various vascular diseases [7]. In another setting, a male mouse model for high running performance recruited myogenic precursor cells/SATCs with lower activation thresholds that responded more rapidly to external stimuli and were more primed for differentiation at the expense of more primitive cells. Satellite cells (SATCs), known as the most abundant skeletal muscle stem cells, play a main role in muscle plasticity, including in the adaptive response following physical activity [8]. In parallel, using pluripotent stem cells (PSCs) to generate hepatocytes is preferable because of their availability and scalability. However, the efficient maturation of PSC-derived hepatocytes towards functional units in liver organoids (LOs) remains a challenging subject. The incorporation of cell-sized microparticles (MPs) derived from the liver extracellular matrix (ECM) provides an enriched tissue-specific microenvironment for the further maturation of hepatocytes inside LOs [9]. This approach has led to the improvement of hepatocyte-like cells in terms of gene expression and function, CYP activities, albumin secretion, and the metabolism of xenobiotics. An experimental basis for the application of stem cells in the treatment of keloids, a pathological scar observed during wound healing, has been developed. Moreover, a co-culture method has been set up to investigate the influence and mechanism of human dental pulp stem cells (HDPSCs) on keloid fibroblast properties [10]. HDPSCs inhibited the migration, the synthesis of the extracellular matrix, and the expression of pro-fibrotic genes within human keloid fibroblasts (HKFs), while promoting the expression of anti-fibrotic genes. Therefore, it can be concluded that HDPSCs can themselves be used as a tool for restraining/hindering the initiation or progression of fibrotic tissue. Mechanistically, new findings have established ten eleven translocation 1 (Tet1) as a regulator of embryonic stem cell (ESC) proliferation by suppressing p21 to ensure a rapid G1-to-S progression [11]. Moreover, Zscan4, which is highly upregulated in telomerase-deficient late-generation mouse ESCs and human alternative lengthening of telomeres (ALT) cancer cells, has been shown to contribute to the telomere maintenance of those cells without telomerase activities [12]. Several features are still to be identified and resolved for improving the safety and efficiency of stem cell-based therapy, in particular for the use of biological delivery systems. Thus, a systematic literature review investigates the potential of therapy with MSCs associated with fibrin glue on the regeneration of the central or peripheral nervous system [13]. Recently, various strategies using a hydrogel-based system, both as encapsulated stem cells and as biocompatible patches loaded with stem cells and applied at the tissue damage site, were developed for regenerating the infarcted myocardium [14]. Joint engineering, representing a potential tool for cartilage regeneration, is an interdisciplinary field that aims to recreate a neo-tissue whose physical and biochemical properties are close to those of the native tissue. It combines cells, biomaterials, and environmental factors. A particular focus on the extrusion bioprinting of cellularized hydrogels for articular cartilage tissue engineering has been discussed [15]. Furthermore, approaches for optimizing standard MSC culture protocols during this essential primary step of in vitro expansion are required. Several studies suggest some improvements in culture media components (amino acids, ascorbic acid, glucose level, growth factors, lipids, platelet lysate, trace elements, serum, and xenogeneic components) as well as culture conditions and processes (hypoxia, cell seeding, and dissociation during passaging) in order to preserve MSC phenotypes and functionality during the primary phase of in vitro culture [16]. Collectively, this Special Issue, managed and supervised by Dr. Mehdi Najar, successfully gathers a great collection of research articles and reviews highlighting recent fundamental and applied advances in different types of stem cells.
